# Dangerous Cosmetics

**Published:** 1883-01

**Authors:** 


### DANGEROUS.

A young woman employed as a dancer in a traveling company of players, died suddenly a week or two ago, killed, the physician said, by the poisoning of her blood from the paints used in making up her face for the stage.

It is known that a famous clown and pantomimist died of softening of the brain, induced by the pigment used to give his face its chalky whiteness.

The ill effects of such applications are not confined to actors, who use them as one of the appliances of their business. Modest young girls "make up" their faces for the ball-room, or the street, whitening the skin, blackening the brows, removing superfluous hair, etc., by means of antimony, bismuth, white lead and other poisonous compounds.

The poisons do not necessarily kill, though sometimes they produce physical conditions that may lead to death; but before middle age they leave the skin dry, yellow and cracked, and induce headaches and dimness of sight.

In the Southwest still more dangerous methods, it is said, are resorted to for the purpose of improving the complexion. Arsenic is often taken habitually, and belladonna is inserted into the eyes in order to enlarge the pupils, although the victim while under its effects is purblind, and runs the risk of becoming blind altogether.

The worst agents in propagating these practices are paragraphs and advertisements in the newspapers, recommending cosmetics, depilatories and anti-fat medicines. A moment's reflection should teach persons who are inclined to use the latter compounds, that a medicine powerful enough to remove the fatty deposits of the body in a week or fortnight, or even in somewhat longer time than that, must also destroy the tissues. Death has resulted from their use, and low fevers are not infrequently produced by them.

It is said that the women in Paris, whose only capital is their beauty, preserve it by rigorous attention to daily bathing, to exercise and to sleep. Let American girls take the hint, regardless of the source from which it comes.—*Youths Companion.*

